# In Vivo Evaluation of Retinal Neurodegeneration in Patients with Multiple Sclerosis

**DOI:** 10.1371/journal.pone.0030922

**Published:** 2012-01-26

**Authors:** Erika Tátrai, Magdolna Simó, Anna Iljicsov, János Németh, Delia Cabrera DeBuc, Gábor Márk Somfai

**Affiliations:** 1 Department of Ophthalmology, Semmelweis University, Budapest, Hungary; 2 Department of Neurology, Semmelweis University, Budapest, Hungary; 3 Bascom Palmer Eye Institute, University of Miami Miller School of Medicine, Miami, Florida, United States of America; Institute Biomedical Research August Pi Sunyer (IDIBAPS) - Hospital Clinic of Barcelona, Spain

## Abstract

**Objective:**

To evaluate macular morphology in the eyes of patients with multiple sclerosis (MS) with or without optic neuritis (ON) in previous history.

**Methods:**

Optical coherence tomography **(**OCT) examination was performed in thirty-nine patients with MS and in thirty-three healthy subjects. The raw macular OCT data were processed using OCTRIMA software. The circumpapillary retinal nerve fiber layer (RNFL) thickness and the weighted mean thickness of the total retina and 6 intraretinal layers were obtained for each eye. The eyes of MS patients were divided into a group of 39 ON-affected eyes, and into a group of 34 eyes with no history of ON for the statistical analyses. Receiver operating characteristic (ROC) curves were constructed to determine which parameter can discriminate best between the non-affected group and controls.

**Results:**

The circumpapillary RNFL thickness was significantly decreased in the non-affected eyes compared to controls group only in the temporal quadrant (p = 0.001) while it was decreased in the affected eyes of the MS patients in all quadrants compared to the non-affected eyes (p<0.05 in each comparison). The thickness of the total retina, RNFL, ganglion cell layer and inner plexiform layer complex (GCL+IPL) and ganglion cell complex (GCC, comprising the RNFL and GCL+IPL) in the macula was significantly decreased in the non-affected eyes compared to controls (p<0.05 for each comparison) and in the ON-affected eyes compared to the non-affected eyes (p<0.001 for each comparison). The largest area under the ROC curve (0.892) was obtained for the weighted mean thickness of the GCC. The EDSS score showed the strongest correlation with the GCL+IPL and GCC thickness (p = 0.007, r = 0.43 for both variables).

**Conclusions:**

Thinning of the inner retinal layers is present in eyes of MS patients regardless of previous ON. Macular OCT image segmentation might provide a better insight into the pathology of neuronal loss and could therefore play an important role in the diagnosis and follow-up of patients with MS.

## Introduction

Multiple sclerosis (MS) is a chronic inflammatory disorder that affects the central nervous system. The disease is characterized by demyelination that leads to axonal dysfunction and neuronal loss [1]. Unmyelinated neuronal axons offer a good possibility to examine axonal loss as the thickness of the myelin sheath does not affect the nerve thickness results. The innermost layer of the retina is the retinal nerve fiber layer (RNFL) being comprised of the axons of the retinal ganglion cells which get myelin sheath only after leaving the eye through the lamina cribrosa. Therefore, the thickness measurement of the RNFL might be a good marker of the axonal damage in MS patients. Optical coherence tomography (OCT) is a non-invasive, non-contact diagnostic tool that provides high-resolution cross-sectional images of the retina [2]. This technique enables, among others, the measurement of the thickness of the RNFL around the optic disc and also the thickness and volume of the macula lutea in vivo. With the use of OCT image processing, not only the thickness of the total retina but also the thickness of the intraretinal layers can be measured in the macular area [3]–[5].

The loss of retinal nerve fibers around the optic disc (circumpapillary RNFL – cpRNFL) has been found in the eyes of MS patients both with and without optic neuritis (ON) in the history [6]–[11]. However, recent studies have shown that also macular thickness and volume are decreased in the eyes of patients with MS [6], [7], [12]–[14], presumably caused by the thinning of the ganglion cell layer (GCL) and inner plexiform layer (IPL) [11], [15]–[17].

The purpose of our study was to assess macular morphology in patients with MS with or without ON in previous history and also to determine which OCT parameter has the greatest ability to detect neuronal damage in patients with MS. We could demonstrate that the thickness of the ganglion cell complex (GCC) is the most sensitive marker for the detection of neuronal loss due to ON and we could also show that there are signs of axonal degeneration even without ON in previous history. The thickness of the GCL+IPL complex and the GCC in the macula showed the strongest correlation with the clinical measure of disability measured by the EDSS score.

## Methods

### Patient population and study design

Thirty-nine patients with relapsing-remitting multiple sclerosis meeting the revised McDonald criteria [18] were consecutively recruited from the Department of Neurology of Semmelweis University between October 2008 and June 2011 in this cross-sectional case-control study. Exclusion criteria for all patients were: (1) spherical or cylindrical correction higher than 3.0 diopters, (2) the presence of any retinal disease or optic neuropathy including glaucoma, except ON, (3) intraocular pressure higher than 20 mmHg in the medical history, (4) previous eye surgery, (5) amblyopia, (6) last ON episode less than 6 months prior to enrollment, (7) bad fixation cooperation during the OCT examination (e.g. due to nystagmus) and (8) low signal strength of the OCT images (SS≤6). Five eyes were excluded from the study due to the presence of retinal disease (1 eye), acute ON (1 eye), low signal strength of the OCT image due to media opacity (1 eye) and amblyopia (2 eyes). Thirty-three randomly selected eyes of thirty-three age-matched controls were also examined with OCT. The eligibility criteria for control subjects were best-corrected Snellen visual acuity of 20/20 and the lack of any ocular or systemic diseases. Demographic and clinical characteristics including age, gender and duration of disease are listed in Table 1.

**Table 1:** Clinical characteristics of the study patients.

Characteristic	Control (n = 33)	MS (n = 39)
Age, mean±SD, y	34.3±8.3	34.0±8.2
Age, median (range)	33 (21–52)	34 (19–53)
No. (%) female	23 (69.7)	27 (69.2)
ON-affected eye, No. (%)	NA	39 (53.0)
Disease duration, mean±SD, y	NA	6.5±3.9

Abbreviations: NA, not applicable; MS, multiple sclerosis; SD, standard deviation.

All participants were treated in accordance with the tenets of the Declaration of Helsinki. Institutional Review Board approval was obtained for all study protocols (Semmelweis University Regional and Institutional Committee of Sciences and Research Ethics). Written informed consent was obtained from all participants in this study.

### Ophthalmic and neurological examinations

**Figure 1:**
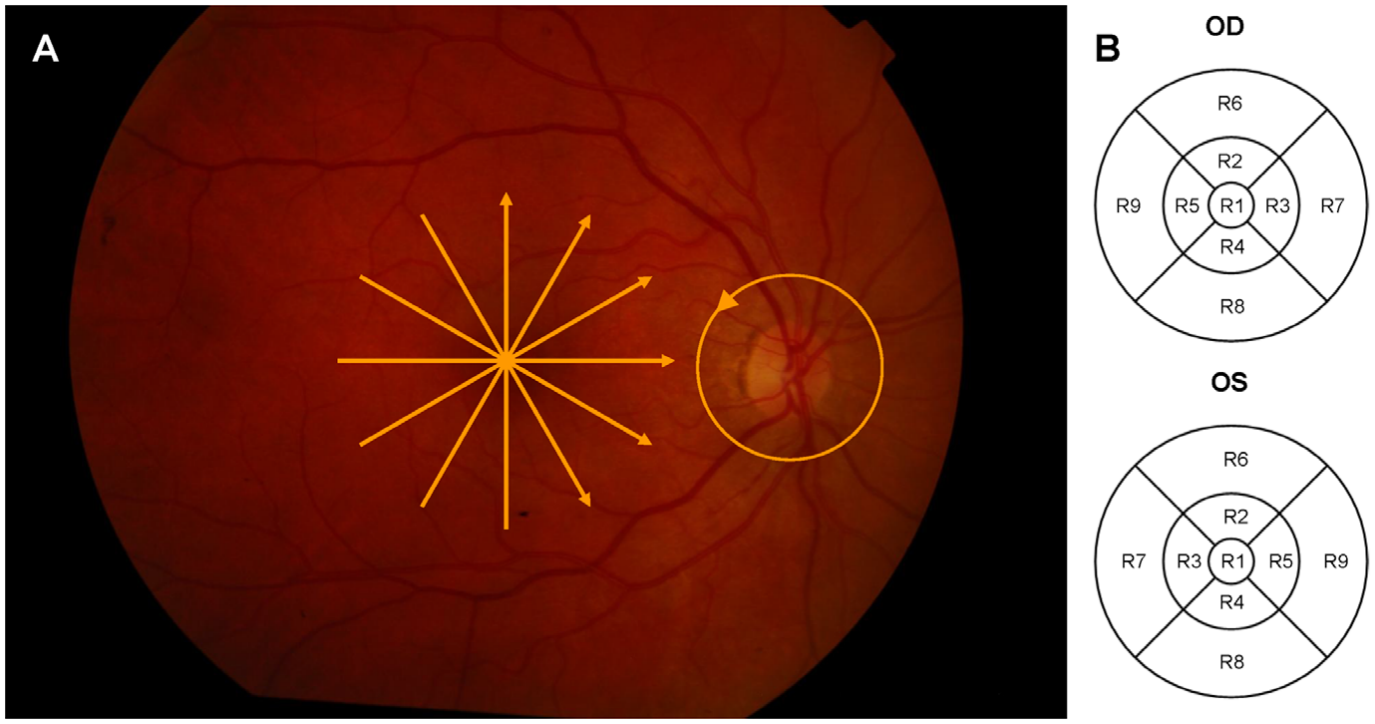
Retinal scanning used in the study. The yellow arrows in the macula and the circle around the optic nerve head show the locations of the OCT scans made. (B) The distribution of ETDRS regions for the right (OD) and left eye (OS).

All patients underwent an ophthalmic examination including best-corrected Snellen visual acuity, assessment of intraocular pressure, slit lamp biomicroscopy and binocular ophthalmoscopy with pupil dilation. The same day, OCT examination was performed on each eye using a Stratus OCT device (Carl Zeiss Meditec, Dublin, CA, USA). “Fast RNFL map protocol” consisting of three circular scans with diameters of 3.4 mm centered on the optic disc was performed (Fig. 1). The mean overall and sectoral (superior, nasal, inferior and temporal) cpRNFL thickness values were recorded for each eye. The mean overall cpRNFL thickness was calculated by averaging the thickness values in the four sectors. To assess the thickness of the intraretinal layers of the macula, each eye was scanned using the “Macular thickness map” protocol consisting of six evenly spaced radial lines centered on the fovea, each having a 6 mm transverse length (Fig. 1).

The patients underwent a comprehensive neurological examination within one week of the ophthalmic examination. To assess physical disability, the Expanded Disability Status Scale (EDSS) score was determined for each patient.

### OCT image processing (OCTRIMA)

**Figure 2:**
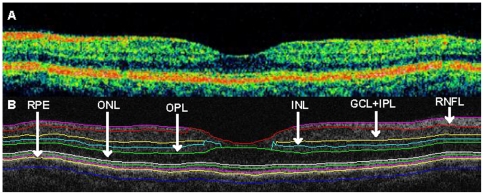
Macular image segmentation using OCTRIMA. The image of a healthy macula scanned by Stratus OCT. (B) The same OCT scan processed with OCTRIMA. We note the ONL segment includes the IS of photoreceptors and the external limiting membrane, which are not resolved by the Stratus OCT device. Abbreviations: GCL+IPL, ganglion cell layer and inner plexiform layer complex; INL, inner nuclear layer; ONL, outer nuclear layer; OPL, outer plexiform layer; RNFL, retinal nerve fiber layer; RPE, retinal pigment epithelium.

The raw macular OCT data were exported from the Stratus OCT device and further processed using a computer-aided grading methodology for OCT retinal image analysis (OCTRIMA) developed by Cabrera et al. [3]. The OCTRIMA software integrates a novel denoising and edge enhancement technique along with a segmentation algorithm. Moreover, the software gives quantitative information of intraretinal structures and facilitates the analysis of other retinal features that may be of diagnostic and prognostic value, such as the morphology and reflectivity by enabling the segmentation of the various cellular layers of the retina [3], [19]. The OCTRIMA software enables the segmentation of 6 layers of the retina on OCT images based on their optical densities: the RNFL, the GCL+IPL complex, the inner nuclear layer (INL), the outer plexiform layer (OPL), the outer nuclear layer (ONL) and retinal pigment epithelium (RPE). We have previously shown the high repeatability and reproducibility of OCTRIMA measurements in normal subjects with undisrupted retinal structure similarly to what is observed in patients with MS [20], [21]. The reproducibility was the highest for the thickness measurements of the ONL, GCC, GCL+IPL and RNFL, the inter- and intraexaminer, intervisit variabilities (<6 µm for all layers and all comparisons) being under the resolution of the Stratus OCT device for all layers [21]. The OCT scan of a healthy macula before and after segmentation with OCTRIMA can be seen on Fig. 2. It is important to note that OCTRIMA measures the thickness of the total retina between the inner limiting membrane and the inner boundary of the photoreceptor outer segment/RPE junction. On the contrary, Stratus OCT measures the thickness of the total retina between the inner limiting membrane and the photoreceptor inner segment-outer segment junction. We also note that the ONL is actually enclosing the external limiting membrane and the inner segment of the photoreceptors but in the standard 10 µm resolution OCT image this thin membrane can not be visualized clearly making the segmentation of the inner segment difficult. Thus this layer classification is our assumption and does not reflect the actual anatomic structure.

### Statistical analysis

The thickness of the total retina and the intraretinal layers were measured in the nine macular regions defined by the Early Treatment Diabetic Retinopathy Study (ETDRS) (Fig. 1) [22]. Since the number of sampling points is different at the central (R1), pericentral (R2–R5) and peripheral (R6–R9) regions because of the radial spoke pattern used in the scanning protocol of Stratus OCT, a weighted mean thickness (WMT) was calculated instead of averaging retinal thickness results in the 9 macular regions [23]. The WMT was generated using the following equation:
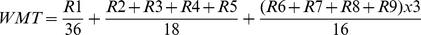



The WMT values for the RNFL, GCL+IPL, GCC, INL, OPL, ONL, RPE and the total retina were obtained for each eye. Since the GCC is composed by the RNFL and GCL+IPL consisting of distal and proximal parts of the retinal ganglion cells, the thickness of the GCC was used to describe the integrity of the ganglion cells.

The correlation between the disease duration, age, EDSS score and the cpRNFL and macular intraretinal thickness parameters was calculated by linear correlation. Furthermore, the correlation between the cpRNFL parameters and the thickness of the intraretinal structures was also determined by linear correlation.

The eyes of the MS patients were divided into two study groups for further analyses. The first group was composed of 39 eyes which had ON at least 6 months prior to enrollment. All eyes in this group had only one previous episode of ON. The second group was composed of 34 eyes which had no history of ON. The diagnosis of optic neuritis was based on the patient's medical history and defined by clinical symptoms such as decreased visual acuity developing in few days, pain on eye movement, abnormal response on visual evoked potential examination confirming prechiasmal lesion and decrease in the critical flicker frequency (CFF). CFF is a routine examination for the assessment of optic nerve function. Particularly, in the context of optic neuritis, the CFF is known to be decreased in the acute phase of ON and also it can show impairment after the recovery. Therefore, measuring the CFF can help to establish the diagnosis.

All measured thickness values were compared among the groups using mixed model ANOVA. Receiver operating characteristic (ROC) curves were constructed to describe the ability of each parameter to discriminate between the eyes of MS patients not affected with ON and the eyes of the control group.

Statistical analyses were performed using Statistica 8.0 (Statsoft Inc., Tulsa, OK, USA) and SPSS 15.0 (SPSS Inc., Chicago, IL, USA). A p value of <0.05 was considered statistically significant.

## Results

All eyes had a Snellen visual acuity of 1.0. The strongest correlation was observed between the EDSS and the GCL+IPL, GCC and mean overall cpRNFL (p = 0.007, p = 0.007 and p = 0.008, respectively; r = −0.43 for all variables) while the correlations with the inferior cpRNFL, superior cpRNFL and the WMT of the RNFL in the macula was weaker (p = 0.02, p = 0.05 and p = 0.05, respectively; r = −0.38, r = −0.33 and r = −0.32, respectively). The remaining intraretinal layers and cpRNFL parameters showed no correlation with the EDSS. There was no correlation between any of the thickness values measured and either disease duration or age.

The mean overall cpRNFL thickness and the cpRNFL thickness in the superior, nasal, inferior and temporal quadrants was significantly decreased in the eyes of MS patients previously affected with ON compared to the non-affected eyes of MS patients (see Table 2). Each cpRNFL thickness parameter was significantly lower in the ON-affected eyes compared to controls except for the cpRNFL thickness in the nasal quadrant. However, the eyes not affected with ON showed significantly lower cpRNFL thickness values compared to the control group only in the temporal quadrant. The cpRNFL was thinner in the superior, nasal, inferior and temporal quadrants by 16%, 11%, 16% and 27%, respectively in the ON-affected eyes compared to controls and by 6%, 4%, 4% and 17%, respectively in the eyes without ON in medical history compared to controls.

**Table 2:** WMT values of the intraretinal layers and the total retina in the study groups.

	Thickness, µm, mean±SD			*p* values		
Intraretinal layer	Control (33 eyes)	NE group (34 eyes)	AE group (39 eyes)	NE vs. Control	AE vs. Control	NE vs. AE
RNFL	38±3	34±3	31±5	0.001*	<0.001*	<0.001*
GCL+IPL	72±4	63±6	54±7	<0.001*	<0.001*	<0.001*
GCC	109±6	98±8	85±12	<0.001*	<0.001*	<0.001*
INL	33±2	34±2	33±2	0.541	0.740	0.622
OPL	32±2	32±2	32±2	0.612	0.721	0.210
ONL	79±5	81±7	81±6	0.196	0.087	0.396
RPE	12±1	12±1	12±1	0.950	0.589	0.390
Total Retina	290±8	283±11	268±14	0.013*	<0.001*	<0.001*
**cpRNFL sector**						
Mean Overall	103±7	94±19	85±14	0.101	<0.001*	0.037*
Superior	131±10	122±21	110±19	0.051	<0.001*	0.001*
Nasal	77±13	81±21	69±17	0.328	0.066	0.002*
Inferior	130±16	124±18	109±21	0.141	<0.001*	<0.001*
Temporal	73±13	61±12	53±13	0.001*	<0.001*	0.002*

*statistically significant.

Abbreviations: SD = standard deviation; NE = non-affected eye; AE = affected eye; RNFL = retinal nerve fiber layer; GCL+IPL = ganglion cell layer and inner plexiform layer complex; GCC = ganglion cell complex; INL = inner nuclear layer; OPL = outer plexiform layer; ONL = outer nuclear layer; RPE = retinal pigment epithelium.

The WMT of the total retina, RNFL, GCL+IPL and GCC showed a significant decrease in both the ON-affected and non-affected eyes of MS patients compared to the control group (Table 2). Furthermore, the eyes previously affected with ON had significantly lower thickness values in these layers than the eyes not affected with ON. There was no statistically significant difference in any of the remaining intraretinal layers among the groups.

The strongest correlation was observed between the mean overall cpRNFL thickness and the thickness of the GCL+IPL and GCC in the macula (r = 0.76 and r = 0.75, respectively) while the correlation was weaker in the case of the total retina in the macula (r = 0.68). An average 10 µm loss of the average cpRNFL thickness was associated with 7.5 µm reduction in the total retinal thickness and also a 7.5 µm reduction in the thickness of the GCC, the latter resulting from a 5.3 µm reduction of the GCL+IPL and a 2.2 µm reduction of the RNFL.

**Figure 3:**
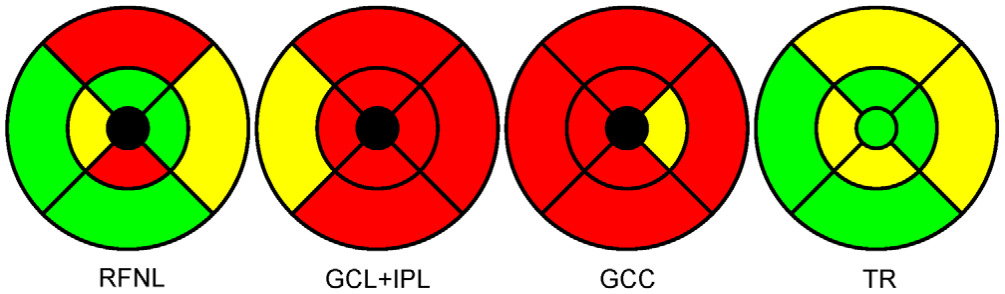
Regional differences between the non-affected eyes of MS patients and healthy eyes. The colors show the extent of thinning based on the p-values of the thickness comparisons. All representative numerical data are in Table 3. The color codes are as follows: red: p<0.001, yellow: 0.001<p<0.05, green: p>0.05. We note the central subfield (R1: black color) was excluded from the analysis for the layers which are not present in the foveal area. Abbreviations: GCC, ganglion cell complex; GCL+IPL, ganglion cell layer and inner plexiform layer complex; RNFL, retinal nerve fiber layer; TR, total retina.

The thickness of the RNFL in the macula was significantly decreased in the non-affected eyes of MS patients compared to the healthy eyes in the inner inferior (R4), inner temporal (R5), outer superior (R6) and outer nasal (R7) regions (see Table 3 and Figure 3). In the eyes affected with ON, macular RNFL thickness was significantly lower in each region compared to the control group and it was also lower compared to the eyes not affected with ON in each region except for the outer temporal region (R9). The thinning of the GCL+IPL was observed in each ETDRS region in the non-affected eyes of MS patients compared to controls and in the ON-affected eyes of MS patients compared to both healthy and non-affected eyes. The thickness of the GCC was found to be significantly lower in each region in the non-affected eyes of MS patients compared to the control group and in the ON-affected eyes compared to both the control group and the non-affected eyes (Table 3 and Figure 3). The thickness of the total retina was significantly thinner in each region except for the central region (R1) in the affected eyes of the MS patients compared to the healthy eyes while it was thinner in each region in the affected eyes compared to the non-affected. For the comparison of the eyes of MS patients not affected with ON versus healthy subjects' eyes there was a significant thinning only in the inner inferior (R4), inner temporal (R5), outer superior (R6) and outer nasal (R7) regions (see Table 3 and Figure 3).

**Table 3:** Regional differences in the thickness of the intraretinal layers in the groups which showed statistically significant difference.

		Thickness, µm, mean±SD			*p* values	
	Control (33 eyes)	NE group (34 eyes)	AE group (39 eyes)	NE vs. Control	AE vs. Control	NE vs. AE
***RNFL***						
R1	NL	NL	NL	NA	NA	NA
R2	27±4	26±4	23±5	0.172	<0.001*	0.002*
R3	24±4	23±4	19±6	0.291	<0.001*	<0.001*
R4	30±4	25±4	21±6	<0.001*	<0.001*	0.001*
R5	19±5	15±4	13±5	0.001*	<0.001*	0.027*
R6	51±5	46±4	41±5	<0.001*	<0.001*	<0.001*
R7	53±6	47±4	42±7	0.001*	<0.001*	<0.001*
R8	40±3	38±2	35±7	0.107	<0.001*	0.001*
R9	28±4	25±4	23±6	0.051	<0.001*	0.091
***GCL+IPL***						
R1	NL	NL	NL			
R2	96±6	87±9	72±11	<0.001*	<0.001*	<0.001*
R3	95±6	87±11	71±10	<0.001*	<0.001*	<0.001*
R4	94±5	84±8	72±9	<0.001*	<0.001*	<0.001*
R5	95±5	85±8	73±10	<0.001*	<0.001*	<0.001*
R6	67±5	57±8	49±9	<0.001*	<0.001*	<0.001*
R7	72±6	61±9	52±10	<0.001*	<0.001*	<0.001*
R8	61±5	54±5	47±7	<0.001*	<0.001*	<0.001*
R9	69±6	63±6	54±7	0.001*	<0.001*	<0.001*
***GCC***						
R1	NL	NL	NL			
R2	124±8	112±11	95±14	<0.001*	<0.001*	<0.001*
R3	120±8	109±12	90±15	0.001*	<0.001*	<0.001*
R4	124±6	109±11	93±14	<0.001*	<0.001*	<0.001*
R5	113±6	100±11	86±13	<0.001*	<0.001*	<0.001*
R6	117±8	103±9	91±13	<0.001*	<0.001*	<0.001*
R7	125±8	109±10	93±15	<0.001*	<0.001*	<0.001*
R8	101±6	92±7	82±11	<0.001*	<0.001*	<0.001*
R9	97±7	89±7	78±10	<0.001*	<0.001*	<0.001*
***Total retina***						
R1	234±17	237±20	226±14	0.773	0.171	<0.001*
R2	324±13	319±12	299±16	0.115	<0.001*	<0.001*
R3	322±13	318±14	298±17	0.231	<0.001*	<0.001*
R4	322±11	312±10	295±15	0.001*	<0.001*	<0.001*
R5	312±12	302±11	288±14	0.002*	<0.001*	<0.001*
R6	293±8	283±13	268±16	0.006*	<0.001*	<0.001*
R7	301±10	290±14	272±18	0.006*	<0.001*	<0.001*
R8	271±7	267±12	254±14	0.103	<0.001*	<0.001*
R9	271±10	265±13	253±12	0.080	<0.001*	<0.001*

For the distribution of the ETDRS regions see Figure 1.

*statistically significant.

Abbreviations: SD = standard deviation; NE = non-affected eye; AE = affected eye; NL = no layer; NA = not applicable; RNFL = retinal nerve fiber layer; GCL+IPL = ganglion cell layer and inner plexiform layer complex; GCC = ganglion cell complex; INL = inner nuclear layer; OPL = outer plexiform layer; ONL = outer nuclear layer; RPE = retinal pigment epithelium.

The largest area under the curve (AUC) value for the discrimination between the non-affected eyes of MS patients and the eyes of the control group was obtained for the WMT of the GCC (0.892) (see Table 4). The AUC values for the thickness data obtained by the built-in software of the Stratus OCT – namely the cpRNFL thickness in the temporal quadrant and the thickness of the total retina – were below that of the GCC, 0.745 and 0.709, respectively. Within the GCC, the largest AUC was obtained for the inner temporal and outer nasal regions (R5 and R7, respectively) (0.867 and 0.851) for the discrimination between non-affected eyes of MS patients and controls (see Table 4).

**Table 4:** Results of the ROC analysis for the WMT thickness variables which showed significant difference between the eyes not affected with ON and controls and for the regional GCC thickness values.

Variable	AUC	Asymptotic 95% CI		Cutoff point	Sensitivity	Specificity
		Lower bound	Upper bound	(µm)		
WMT values of the intraretinal layers						
GCC	0.892	0.818	0.966	104	76%	74%
GCL+IPL	0.869	0.785	0.952	68	79%	77%
RNFL	0.809	0.707	0.911	35	82%	56%
Temporal cpRNFL	0.745	0.628	0.861	67	73%	68%
Total Retina	0.709	0.585	0.834	288	73%	62%
Regional thickness values for the GCC						
R2 (inner superior)	0.792	0.683	0.902	91	85%	62%
R3 (inner nasal)	0.740	0.616	0.863	91	76%	65%
R4 (inner inferior)	0.845	0.751	0.938	91	79%	77%
R5 (inner temporal)	0.867	0.778	0.957	90	82%	74%
R6 (outer superior)	0.839	0.747	0.931	63	76%	74%
R7 (outer nasal)	0.851	0.764	0.939	65	88%	65%
R8 (outer inferior)	0.820	0.720	0.920	57	79%	62%
R9 (outer temporal)	0.731	0.612	0.850	65	64%	59%

Note that the central region for the GCC is missing due to the partial lack of ganglion cells in the foveola.

Abbreviations: AUC = area under the curve; CI = confidence interval; GCC = ganglion cell complex; GCL+IPL = ganglion cell layer and inner plexiform layer complex; RNFL = retinal nerve fiber layer, cpRNFL = circumpapillary RNFL.

## Discussion

This study evaluated the usefulness of macular OCT image segmentation in patients with MS in order to determine the structural changes of the retina of MS patients. Furthermore, the parameter which could discriminate best the eyes of healthy subjects from the eyes of MS patients was determined.

Objective markers might be necessary not only for the diagnosis but also the follow-up of neuronal damage in MS, which could help to determine the effect of any possible therapeutical interventions in the future as well. Our results showed that the thickness of the macular ganglion cell complex had the highest sensitivity and specificity to detect axonal loss independent of optic neuritis, outperforming the cpRNFL thickness data provided by the analysis software of the commercially available Stratus OCT device. A strong correlation with disease severity measured by the EDSS score was also obtained in the case of the GCC thickness which implies that this parameter might also be useful in the estimation of disease progression as a surrogate marker. Indeed, Syc et al. have used a similar segmentation methodology to ours extracting four retinal layers (after collapsing the INL+OPL layers) in their recent report and found that the thickness of the GCL+IPL could be a valuable parameter for the longitudinal follow-up of neuronal loss after optic neuritis [17]. Our study has also shown that optic neuritis is followed by a targeted loss of ganglion cells in the macula which can also be objectively assessed by quantitative analysis of OCT macular images.

Several studies have reported the atrophy of the RNFL around the optic disc in patients with MS with and even without optic neuritis in medical history [6]–[11]. Our findings confirmed that the mean overall cpRNFL thickness and the cpRNFL thickness in each quadrant is significantly lower in the eyes of MS patients with a history of ON compared to the non-ON-affected eyes and also in comparison with healthy eyes except for the nasal quadrant. However, our results showed that in the non-affected eyes of MS patients the cpRNFL thickness is decreased only in the temporal quadrant compared to healthy eyes. Furthermore, the most pronounced reduction in the thickness of cpRNFL (27% and 17% in the ON-affected and non-affected eyes, respectively) was also observed in the temporal quadrant. These findings are in agreement with previous OCT studies confirming that the fibers of the papillomacular bundle are the most susceptible to damage in ON [9], [24]. One important aspect of this fact, as it has been widely reported in glaucomatous damage, is that the evaluation of the sectoral thickness values could provide the possibility to discriminate the RNFL atrophy caused by glaucomatous damage and other disorders affecting the optic nerve, such as MS [6].

As a result of neuronal loss, not only the thickness of the cpRNFL is decreased but also the macula was found to be thinner in the eyes of MS patients in previous reports [6], [7], [12]–[14]. Histopathological studies had qualitatively shown the atrophy of the inner retina in the eyes of MS patients, while atrophy of the outer nuclear layer was not detected [25], [26]. However, no quantitative measurements were performed because of technical difficulties, e.g. the partial post-mortem detachment occurring in the retina in many of the eyes. Lately, some studies evaluating a low number of patients and using OCT technology showed that the thickness of the inner retinal layers is decreased in the eyes of MS patients [11], [15]–[17]. However, the reliability of the methodologies used in these studies is not known. Burkholder et al. analyzed a large sample consisting of 530 subjects with RRSM, assessing the volume of the total retina in the inner and outer ETDRS rings (also referred to as pericentral and peripheral macular rings). Their results showed the thinning of the inner and outer retinal ETDRS rings in the eyes of MS patients; however, the local morphological changes of the observed thinning could not be identified as they did not use any segmentation methodology. The OCT image segmentation methodology used in our study allowed the quantification of local retinal changes in patients with MS in vivo. Our results confirmed that the atrophy of the RNFL, GCL+IPL and consequently the GCC is present in the macula of patients with MS even in eyes without ON in previous history. Furthermore, we demonstrated that the outer layers of the retina are not involved in this process. Although it was not an inclusion criterion, all patients had only one episode of ON in the history; therefore, the observed changes were not biased by the number of ON episodes. The thinning of the retina was most pronounced in the inner inferior, inner temporal, outer superior and outer nasal regions.

The average cpRNFL thickness showed the strongest correlation with the thickness of the GCL+IPL and GCC in the macula while a weaker correlation was observed with the thickness of the total retina. Previous studies have also shown that there is a correlation between the mean overall cpRNFL thickness and the thickness of the total retina in the macula, the correlation coefficient in the study of Fjeldstad et al. was the same as in our results (r = 0.68) [7]. In the study of Burkholder et al., 10 µm reduction in the mean overall cpRNFL thickness was associated with a 0.2 mm^3^ reduction of the total macular volume [13]. As the area of the macula – which is imaged using Stratus OCT – is approximately 28.26 mm^2^, the volume of 0.2 mm^3^ consequently equals to about 7.1 µm in thickness. Our results have shown a similar amount of reduction (7.5 µm) in the total retinal thickness which was accounted for by the reduction of the GCC thickness.

Previously, the mean overall cpRNFL thickness was found to correlate significantly with functional parameters such as EDSS score and contrast sensitivity [27]–[29]. However, the thickness of the GCL+IPL in the macula was found to correlate better with these functional parameters, which might thus be a better marker of axonal damage [16]. Our results showed good correlation between the EDSS score and the mean overall cpRNFL thickness and also the thickness of the GCL+IPL and the GCC in the macula. However, the ROC analysis revealed that the value most capable of determining the presence of neuronal damage was the weighted mean thickness of the GCC having an AUC value of 0.892 with a cutoff value of 104 µm having the highest sensitivity and specificity. The thickness of the RNFL and GCL+IPL separately showed lower AUCs than the GCC which could be explained with the better reproducibility of the GCC due to the high contrast between the IPL and INL layers. Furthermore, the AUC values observed for the parameters which can be measured using conventional OCT softwares (i.e. the temporal cpRNFL thickness and the average thickness of the total retina expressed as macular volume) were much lower compared to that of the GCC. Despite their lower AUC values we believe these parameters may still be of clinical importance due to their widespread access by all OCT devices. Among the regional thickness values, the thickness of the GCC in the inner temporal region was observed to have the largest AUC value, namely 0.867 (see Table 4).

Although our results showed that the weighted mean GCC thickness may provide a sensitive tool for the assessment of axonal degeneration, care should be taken when interpreting its value as numerous neurodegenerative disorders, such as glaucoma [30]–[34], Alzheimer's disease [35], [36] or Parkinson's disease [37]–[39] may also lead to ganglion cell death. The use of regional values could help the differential diagnosis between various forms of neurodegeneration, as glaucoma could presumably lead to an infero-superior pattern of GCC loss in the macula, while according to our results MS is rather leading to a horizontal loss of the GCC most probably due to the loss of the papillo-macular nerve bundle. However, further research is warranted to justify the above hypothesis.

A potential limitation of the study is the relatively low number of MS patients included. Although the size of our study is in accordance with previous similar reports, a larger set of patients may be desired to obtain information with even higher power. Another weakness might be the time-domain OCT technology used which could both limit the accuracy of our measurements due to lower resolution when compared with spectral-domain OCT technology which facilitates wider and finer sampling of the macular regions. However, we have previously shown the high reliability and reproducibility of OCTRIMA measurements obtained with time-domain OCT [20], [21] and even its comparability with higher resolution Fourier-domain OCT measurements [40].

Our results imply that mainly the ganglion cells are affected in MS and changes can be already present in eyes without previous history of ON which could be the result of axonal loss due to the disease process of MS or mild optic neuritis events not accompanied by pain. By the use of OCT image segmentation, we could also show in vivo that the neuronal damage affects the ganglion cells and not the outer retina, while episodes of ON are resulting in a further pronounced loss of the retinal ganglion cells. Furthermore, our measurements obtained with a custom-built software were shown to be more sensitive compared to standard measurements extracted by the Stratus OCT device (e.g. cpRNFL, total macular volume) and also showed a stronger correlation with physical disability measured by the EDSS. This implies the potential clinical usefulness of the quantification of the macular GCC thickness by OCT image segmentation, which could also facilitate the cost-effective follow-up of neuronal damage due to MS.

In conclusion, we consider that macular OCT image segmentation showing in vivo structural changes of retinal tissue will yield a better insight into macular pathology and therefore should play an important role in the future of the diagnosis and follow-up of neurological diseases affecting the optic nerve, such as multiple sclerosis which influences a continuously increasing number of patients worldwide.

## References

[1] Silber E, Sharief MK (1999). Axonal degeneration in the pathogenesis of multiple sclerosis.. J Neurol Sci.

[2] Huang D, Swanson EA, Lin CP, Schuman JS, Stinson WG (1991). Optical coherence tomography.. Science.

[3] Cabrera Fernandez D, Salinas HM, Puliafito CA (2005). Automated detection of retinal layer structures on optical coherence tomography images.. Opt Express.

[4] Ishikawa H, Stein DM, Wollstein G, Beaton S, Fujimoto JG (2005). Macular segmentation with optical coherence tomography.. Invest Ophthalmol Vis Sci.

[5] Shahidi M, Wang Z, Zelkha R (2005). Quantitative thickness measurement of retinal layers imaged by optical coherence tomography.. Am J Ophthalmol.

[6] Bock M, Brandt AU, Dorr J, Kraft H, Weinges-Evers N (2010). Patterns of retinal nerve fiber layer loss in multiple sclerosis patients with or without optic neuritis and glaucoma patients.. Clin Neurol Neurosurg.

[7] Fjeldstad C, Bemben M, Pardo G (2011). Reduced retinal nerve fiber layer and macular thickness in patients with multiple sclerosis with no history of optic neuritis identified by the use of spectral domain high-definition optical coherence tomography.. J Clin Neurosci.

[8] Parisi V, Manni G, Spadaro M, Colacino G, Restuccia R (1999). Correlation between morphological and functional retinal impairment in multiple sclerosis patients.. Invest Ophthalmol Vis Sci.

[9] Pueyo V, Martin J, Fernandez J, Almarcegui C, Ara J (2008). Axonal loss in the retinal nerve fiber layer in patients with multiple sclerosis.. Mult Scler.

[10] Sepulcre J, Murie-Fernandez M, Salinas-Alaman A, Garcia-Layana A, Bejarano B (2007). Diagnostic accuracy of retinal abnormalities in predicting disease activity in MS.. Neurology.

[11] Tegetmeyer H, Kühn E (2011). Quantitative Analysis of Changes in Macular Layers Following Optic Neuritis.. Neuro-Ophthalmology.

[12] Almarcegui C, Dolz I, Pueyo V, Garcia E, Fernandez FJ (2010). Correlation between functional and structural assessments of the optic nerve and retina in multiple sclerosis patients.. Neurophysiol Clin.

[13] Burkholder BM, Osborne B, Loguidice MJ, Bisker E, Frohman TC (2009). Macular volume determined by optical coherence tomography as a measure of neuronal loss in multiple sclerosis.. Arch Neurol.

[14] Saidha S, Syc SB, Ibrahim MA, Eckstein C, Warner CV (2011). Primary retinal pathology in multiple sclerosis as detected by optical coherence tomography.. Brain.

[15] Davies EC, Galetta KM, Sackel DJ, Talman LS, Frohman EM (2011). Retinal ganglion cell layer volumetric assessment by spectral-domain optical coherence tomography in multiple sclerosis: application of a high-precision manual estimation technique.. J Neuroophthalmol.

[16] Saidha S, Syc SB, Durbin MK, Eckstein C, Oakley JD (2011). Visual dysfunction in multiple sclerosis correlates better with optical coherence tomography derived estimates of macular ganglion cell layer thickness than peripapillary retinal nerve fiber layer thickness.. Mult Scler.

[17] Syc SB, Saidha S, Newsome SD, Ratchford JN, Levy M (2011). Optical coherence tomography segmentation reveals ganglion cell layer pathology after optic neuritis.. Brain.

[18] Polman CH, Reingold SC, Edan G, Filippi M, Hartung HP (2005). Diagnostic criteria for multiple sclerosis: 2005 revisions to the “McDonald Criteria”.. Ann Neurol.

[19] Cabrera DeBuc D, Somfai GM (2010). Early detection of retinal thickness changes in diabetes using Optical Coherence Tomography.. Med Sci Monit.

[20] Debuc DC, Salinas HM, Ranganathan S, Tatrai E, Gao W (2010). Improving image segmentation performance and quantitative analysis via a computer-aided grading methodology for optical coherence tomography retinal image analysis.. J Biomed Opt.

[21] DeBuc DC, Somfai GM, Ranganathan S, Tatrai E, Ferencz M (2009). Reliability and reproducibility of macular segmentation using a custom-built optical coherence tomography retinal image analysis software.. J Biomed Opt.

[22] (1991). Early Treatment Diabetic Retinopathy Study design and baseline patient characteristics. ETDRS report number 7.. Ophthalmology.

[23] Massin P, Vicaut E, Haouchine B, Erginay A, Paques M (2001). Reproducibility of retinal mapping using optical coherence tomography.. Arch Ophthalmol.

[24] Klistorner A, Arvind H, Nguyen T, Garrick R, Paine M (2009). Multifocal VEP and OCT in optic neuritis: a topographical study of the structure-function relationship.. Doc Ophthalmol.

[25] Green AJ, McQuaid S, Hauser SL, Allen IV, Lyness R (2010). Ocular pathology in multiple sclerosis: retinal atrophy and inflammation irrespective of disease duration.. Brain.

[26] Kerrison JB, Flynn T, Green WR (1994). Retinal pathologic changes in multiple sclerosis.. Retina.

[27] Costello F, Hodge W, Pan YI, Eggenberger E, Freedman MS (2010). Using retinal architecture to help characterize multiple sclerosis patients.. Can J Ophthalmol.

[28] Fisher JB, Jacobs DA, Markowitz CE, Galetta SL, Volpe NJ (2006). Relation of visual function to retinal nerve fiber layer thickness in multiple sclerosis.. Ophthalmology.

[29] Henderson AP, Trip SA, Schlottmann PG, Altmann DR, Garway-Heath DF (2008). An investigation of the retinal nerve fibre layer in progressive multiple sclerosis using optical coherence tomography.. Brain.

[30] Garas A, Vargha P, Hollo G (2011). Diagnostic accuracy of nerve fibre layer, macular thickness and optic disc measurements made with the RTVue-100 optical coherence tomograph to detect glaucoma.. Eye (Lond).

[31] Kim NR, Hong S, Kim JH, Rho SS, Seong GJ (2011). Comparison of Macular Ganglion Cell Complex Thickness by Fourier-Domain OCT in Normal Tension Glaucoma and Primary Open-Angle Glaucoma.. J Glaucoma.

[32] Kim NR, Lee ES, Seong GJ, Kim JH, An HG (2010). Structure-function relationship and diagnostic value of macular ganglion cell complex measurement using Fourier-domain OCT in glaucoma.. Invest Ophthalmol Vis Sci.

[33] Schulze A, Lamparter J, Pfeiffer N, Berisha F, Schmidtmann I (2011). Diagnostic ability of retinal ganglion cell complex, retinal nerve fiber layer, and optic nerve head measurements by Fourier-domain optical coherence tomography.. Graefes Arch Clin Exp Ophthalmol.

[34] Tan O, Chopra V, Lu AT, Schuman JS, Ishikawa H (2009). Detection of macular ganglion cell loss in glaucoma by Fourier-domain optical coherence tomography.. Ophthalmology.

[35] Paquet C, Boissonnot M, Roger F, Dighiero P, Gil R (2007). Abnormal retinal thickness in patients with mild cognitive impairment and Alzheimer's disease.. Neurosci Lett.

[36] Parisi V, Restuccia R, Fattapposta F, Mina C, Bucci MG (2001). Morphological and functional retinal impairment in Alzheimer's disease patients.. Clin Neurophysiol.

[37] Altintas O, Iseri P, Ozkan B, Caglar Y (2008). Correlation between retinal morphological and functional findings and clinical severity in Parkinson's disease.. Doc Ophthalmol.

[38] Hajee ME, March WF, Lazzaro DR, Wolintz AH, Shrier EM (2009). Inner retinal layer thinning in Parkinson disease.. Arch Ophthalmol.

[39] Inzelberg R, Ramirez JA, Nisipeanu P, Ophir A (2004). Retinal nerve fiber layer thinning in Parkinson disease.. Vision Res.

[40] Tatrai E, Ranganathan S, Ferencz M, Debuc DC, Somfai GM (2011). Comparison of retinal thickness by Fourier-domain optical coherence tomography and OCT retinal image analysis software segmentation analysis derived from Stratus optical coherence tomography images.. J Biomed Opt.

